# Balancing Equity and Global Health Security Towards a Fair and Effective Pandemic Agreement

**DOI:** 10.3389/ijph.2025.1608581

**Published:** 2025-04-17

**Authors:** Elil Renganathan, Fabrizio Tediosi, Ana Abecasis, Quique Bassat, Astrid Berner-Rodoreda, Nuria Casamitjana, Guenter Froeschl, Simone Kashima, Antoni Placencia, Mario Raviglione, Alberto Rocamora, Jolene Skordis

**Affiliations:** ^1^ Jeffrey Cheah School of Medicine and Health Sciences, Monash University, Bandar Sunway, Malaysia; ^2^ Centre for MultidisciplinAry ResearCh in Health Science (MACH), University of Milan, Milan, Italy; ^3^ Global Health and Tropical Medicine, Institute of Hygiene and Tropical Medicine, NOVA University of Lisbon, Lisbon, Portugal; ^4^ Barcelona Institute for Global Health (ISGlobal), Barcelona, Spain; ^5^ Heidelberg Institute of Global Health, Heidelberg University Hospital, Heidelberg, Germany; ^6^ Department of Infectious Diseases and Tropical Medicine, Ludwig-Maximilians-Universität München (LMU), Munich University Hospital, Munich, Germany; ^7^ University of São Paulo, Ribeirão Preto, Brazil; ^8^ Faculty of Population Health Sciences, Institute for Global Health, University College London, London, United Kingdom

**Keywords:** pandemic preparedness, global health, global health diplomacy, global health governance, global health policy

## Introduction

The COVID-19 pandemic exposed vulnerabilities in health systems and stark inequities between high- and low-income countries in accessing life-saving resources, hindering global control efforts. Recent outbreaks of mpox, Marburg virus disease (MVD) and avian influenza (H5N1) are further reminders of the continuing threat posed by zoonotic diseases. World Health Organization (WHO) Member States agreed in December 2021 to develop a new international instrument for pandemic prevention, preparedness, and response. This agreement aims to strengthen health systems, ensure equitable access to vaccines and treatments, improve supply chains, and foster global collaboration [1].

WHO member state negotiations face barriers, particularly on intellectual property (IP) rights, equitable vaccine distribution, and pathogen sample sharing, with tensions between HICs and LMICs over resource allocation and benefit sharing. Some countries prioritize the amended 2024 International Health Regulations (IHR) as a binding mechanism for pandemic response [2]. However, the IHR lack provisions for fair Pathogen Access and Benefit Sharing (PABS) and a One Health approach, necessitating a distinct pandemic agreement. The slow pace of negotiations has raised concerns about whether a comprehensive agreement will be finalized in time to mitigate the impact of future pandemics [3]. In addition, the recent United States Government decision to withdraw from WHO and to drastically cut development assistance is likely to further threaten successful negotiations and implementation.

## Equity as the Cornerstone of the Pandemic Agreement

The proposed pandemic agreement centers on equity, ensuring universal access to vaccines, diagnostics, therapeutics, personal protective equipment (PPE), and robust health systems. Disparities in COVID-19 vaccine distribution—where high income countries (HICs) secured most doses while low income countries (LICs) faced severe shortages—highlight the need for an agreement that mitigates such inequities. To prevent two-tiered mechanisms which may hamper global recovery in future pandemics, equity commitments must be legally binding and enforceable.

## A Fair PABS Mechanism

PABS remains a key point of contention in negotiations. HICs advocate for unrestricted pathogen sample access to expedite vaccine and therapeutic development, while many LMICs demand binding commitments on benefit sharing. Historically, LMICs have provided pathogen samples without equitable compensation, with resulting innovations disproportionately benefiting wealthier nations. To rectify these disparities, the pandemic agreement must establish a robust PABS mechanism. A structured protocol should enforce clear timelines for pathogen sharing and equitable benefit distribution. This framework could allow non-signatory states to participate in benefit-sharing provisions, fostering broader engagement in pandemic preparedness. Additionally, technology transfer, capacity building, and intellectual property-sharing must be integral to ensure LMICs transition from aid recipients to active contributors in global health innovation. Without these safeguards, the treaty risks failing to facilitate pathogen sample access and thus pandemic recovery, while reinforcing past inequities.

## One Health and Pragmatic Implementation

The proposed pandemic agreement emphasizes One Health—a comprehensive framework that acknowledges the interconnectedness of human, animal, and environmental health—as an essential strategy for pandemic prevention [4]. The majority of emerging infectious diseases, including COVID-19, originate from zoonotic sources. Effective prevention, therefore, requires addressing the root causes of these outbreaks by strengthening surveillance systems at the human-animal-environment interface and enhancing coordination among public health, veterinary, and environmental agencies. Such an integrated approach is critical for identifying potential threats early and preventing local outbreaks from escalating into epidemics and pandemics. However, implementing the One Health approach presents challenges, particularly for LMICs that frequently contend with resource-constrained monitoring systems. Many LMICs lack the infrastructure and technical capacity necessary for the sophisticated surveillance systems envisioned by the agreement.

To realise One Health, the agreement should endorse pragmatic, incremental strategies aligned with LMICs' surveillance capacities. HICs must commit financial and technical support to strengthen surveillance and response systems worldwide, extending beyond health to environmental and planetary health determinants of zoonoses. A phased approach enhances feasibility, sustainability, and interoperability of surveillance systems, fostering global monitoring capacity.

## Sustainable Long-Term Public and Private Financing

A key challenge in pandemic agreement negotiations is securing sustainable, equitable financing and access to essential pandemic-related products. The lack of consensus on patent waivers and referral of the issue to the World Trade Organization (WTO) is regrettable. The economic costs of inaction during COVID-19 far exceeded those of preparedness [5, 6], yet global financing for pandemic prevention, preparedness, and health system resilience remains fragmented and inadequate [7]. Amid competing global priorities, pandemic preparedness—an essential global public good—requires strengthened, predictable funding. Although the amended IHR [2] include a financial coordination mechanism, further efforts are needed to ensure LMICs access necessary resources for effective pandemic response.

A key priority of the pandemic agreement should be establishing a unified, sustainable financing structure that supports both the IHR and the broader objectives of the pandemic agreement. This financing mechanism must ensure long-term, predictable funding to enable LMICs to strengthen health systems and enhance surveillance capacities, thereby bolstering pandemic prevention, preparedness, response and resilience. The pandemic agreement should incentivize push-and-pull mechanisms to prioritize global public goods, ensuring equitable access to critical technologies like vaccines and medicines. The growing emphasis on Environmental, Social, and Governance (ESG) standards [8] presents an opportunity to redefine the private sector’s role in global health. Aligning with ESG principles fosters ethical engagement in pandemic preparedness, enabling the private sector to support a resilient and sustainable global health system.

## Implementation, Accountability, and Transparency

Even the best designed agreement will fail if not properly implemented and enforced. A key debate in the pandemic agreement negotiations is whether to include formal compliance mechanisms, as such mechanisms may be politically difficult to implement.

One possible solution is the creation of an implementation committee, similar to the one that will be established under the IHR amendments [9]. This committee could oversee the implementation of both the IHR and the pandemic treaty, ensuring coherence between the two. The committee could also play a crucial role in monitoring compliance, particularly with regard to equity and benefit-sharing commitments.

In addition to oversight, the agreement should include provisions for transparency and civil society engagement and active participation. Countries should be required to report regularly on their progress to the WHO in implementing the agreement’s provisions, and these reports should be made publicly available to allow for independent scrutiny. Transparency can serve as a powerful tool for accountability, even in the absence of formal compliance mechanisms.

## Conclusion

With experts estimating a 28% probability of a global influenza or coronavirus pandemic resulting in at least one million deaths within the next 5 years [10], it is crucial to recognize the intrinsic link between global security and equity. Mutual trust forms the foundation of effective responses to future global health crises, underscoring the need for cooperative, equitable approaches that strengthen preparedness and resilience worldwide [11]. The current United States Government intention to sever relations with WHO threatens global equity efforts in pandemic preparedness.

The ongoing negotiations for a pandemic agreement represent a critical opportunity for signatory countries to consolidate their alliance and create a more resilient global health system. For this treaty to succeed, equity must be at its foundation, necessitating substantive compromises from all stakeholders. HICs must make firm commitments to support equitable access to pathogens, benefit-sharing and sustainable financing mechanisms. At the same time, MICs will need to shift to domestic financing for sustainable preparedness, while LICs will continue to need external support to implement One Health approaches to strengthen their health and surveillance systems.
